# A Comparative Study of Bacterial Infections Between COVID-19 and Non-COVID-19 Patients With Respect to Different Isolates and Their Antibiotic Sensitivity Pattern

**DOI:** 10.7759/cureus.40387

**Published:** 2023-06-13

**Authors:** Nitali Arun, Kumar Saurabh, Sweta Muni, Namrata Kumari, Anand Dev

**Affiliations:** 1 Microbiology, Indira Gandhi Institute of Medical Sciences, Patna, IND; 2 Emergency Medicine, Indira Gandhi Institute of Medical Sciences, Patna, IND

**Keywords:** antimicrobial sensitivity, multidrug resistant (mdr), ast pattern, amr surveillance, covid-19

## Abstract

Background

Following the pandemic caused by SARS-CoV-2, the emergence of and following the pandemic has required major modifications to healthcare systems and frameworks. Antimicrobials have more than a few potential roles in managing COVID-19. Experimental cures for the treatment of SARS-CoV-2 are being explored. The availability of limited data suggests that nosocomial infections are associated with a higher risk of death and severity of COVID-19. To fill this knowledge gap, we conducted a study to assess the spectrum of bacteriological isolates in different samples from COVID-19 patients. Our study aimed to evaluate the antibiotic resistance pattern of these bacterial isolates and compare the spectrum of bacteriological isolates in different samples and their antibiotic sensitivity pattern between COVID-19 and non-COVID-19 patients.

Methodology

An observational cross-sectional, partly retrospective, and partly prospective study was carried out in the bacteriology section of the Department of Microbiology, Indira Gandhi Institute of Medical Sciences (IGIMS), Patna, Bihar, for a total duration of six months from February 2021 to July 2021. The profile of pathogens isolated from 105 clinical samples from COVID-19 patients was studied. To detect COVID-19, RT-PCR was performed. All clinical specimens (urine, blood, pus, respiratory sample, etc.) were processed and cultured on different media to support the growth of the bacteria as per our standard operating procedures (SOP) for bacteriology samples. Antimicrobial susceptibility testing was carried out based on the Clinical and Laboratory Standards Institute.

Results

A total of 105 clinical samples were received in the bacteriology section of the Department of Microbiology, IGIMS, Patna, Bihar, from admitted COVID-19 patients. The mean age of study participants was 51.57 ± 14.76 years, and males (66.7%, 70/105) were more than females (33.3%, 35/105). The majority of the patients were 91 out of 105 (86.67%) from the ward and 14 from the ICU (13.33%). Of the total samples tested, 62 (59%) were urine samples, 26 (24.8%) were respiratory specimens, 13 (12.4%) were pus samples, 3 (2.9%) were body fluids, and 1 (1%) were tissue samples. Among the total pathogen isolates (n=57) obtained from patients with SARS-CoV-2 admitted to the ward and ICU, 56.14% (32) were gram-negative, 26.31% (15) were *Candida*, and 17.54% (10) were gram-positive pathogens. The most isolated pathogen was *Escherichia coli* (39.02%, 16/41) followed by *Klebsiella pneumoniae* (29.26%, 12/41), *Acinetobacter baumannii* (7.31%, 3/41), and *Enterobacter cloacae* (2.43%, 1/41). *Enterococcus *spp. as gram-positive bacteria were isolated in 21.95% (9/41) of patients. Among the gram-negative bacteria (*Enterobacterales*), the highest resistance was seen in ampicillin (100%,29/29). For non-*Enterobacterales*, the highest resistance was seen in ceftriaxone (66.66%,2/3).* Enterococcus *spp. showed maximum ciprofloxacin, and gentamicin (high level) resistance was 100% (9/9).

Conclusion

Secondary infections with resistant pathogens in COVID-19 patients highlight the importance of antimicrobial management programs focused on the optimal selection of empirical treatments based on culture reports.

## Introduction

A novel coronavirus, also known as SARS-CoV-2 (also called COVID-19 and 2019-nCoV), was first reported in Wuhan, Hubei Province, China, in December 2019. Since then, the virus has not only been spreading worldwide but has also claimed millions of lives. Because of serious respiratory disease in humans due to SARS-CoV-2, some patients need to be hospitalized, and in severe cases, ICU with mechanical ventilation support is essential (~ 5-15%) [1,2]. The emergence of and following the pandemic caused by SARS-CoV-2 has required major modifications to healthcare systems and frameworks [3-5]. As per recent studies, bacterial co-infection upon admission has been reported in 3.1-3.5% of COVID-19 patients, while secondary bacterial infections, following hospitalization, occurred in up to 15% of patients [6-8].

Antimicrobials have more than a few potential roles in managing COVID-19) Experimental cures for the treatment of SARS-CoV-2 are being explored [9]. The availability of limited data suggests that nosocomial infections are associated with a higher risk of death and severity of COVID-19 [10,11]. Moreover, antibiotic resistance among the microorganism causing secondary infections is also a hidden threat lurking behind COVID-19 patients. Many hospital-acquired infections (HAIs) such as hospital/ventilator-associated pneumonia, bloodstream infection, and UTI may go unreported due to non-culture practices/lack of culturing facilities in many hospitals. More research is needed to determine whether COVID-19 patients are at a higher risk of infection at certain sites or from specific agents. [12].

There is a serious risk of antimicrobial resistance (AMR) aggravating in the COVID-19 pandemic due to the limited treatment options available and the risk that empiric antimicrobial treatment will increase secondary and co-infections. Studies have shown that infections secondary to COVID-19 can be more likely to arise in patients with COVID-19. Hospitalization of COVID-19 cases predisposes them to undesirable consequences, the most serious of which are HAIs/secondary infections [12].

According to our indigenous AMR data, the Indian Council of Medical Research has developed guidelines for treating HAIs. As part of antimicrobial stewardship programs, appropriate empirical treatments and rapid de-escalation must be supported based on culture data. To guide evidence-based COVID-19 treatment, clinical data on viral, bacterial, and fungal infections are needed globally and country by country.

To fill this knowledge gap, we conducted a partly retrospective and partly prospective observational cross-sectional study to assess the spectrum of bacteriological isolates in different samples from COVID-19 patients. Our study aimed to evaluate the antibiotic resistance pattern of these bacterial isolates and compare the spectrum of bacteriological isolates in different samples and their antibiotic sensitivity pattern between COVID-19 and non-COVID-19 patients.

## Materials and methods

Study design

An observational cross-sectional, partly retrospective, and partly prospective study was carried out in the bacteriology section of the Department of Microbiology, Indira Gandhi Institute of Medical Sciences (IGIMS), Patna, Bihar, for a total duration of six months from February 2021 to July 2021. In this study, we included samples that we received in our bacteriology laboratory for culture and sensitivity from COVID-19-positive patients, and retrospectively, we randomly included data from COVID-19-negative patients who were culture positive. The study included 105 COVID-19-positive patients who were admitted to the hospital during the study period and gave consent to participate. The study included blood, urine, respiratory samples, pus, and other samples from COVID-19 and non-COVID-19 patients admitted to the hospital. Patients below the age of 18 years were excluded from the study.

A pretested structured questionnaire was used for data collection. The study participants were interviewed about their sociodemographic details (only those who were positive for COVID-19), while the patients' COVID-19 reverse transcriptase polymerase chain reaction (RT-PCR) status, sample details, isolates, and antibiotic sensitivity testing (AST) data were noted from the laboratory records of the Molecular and Bacteriology sections of the Department of Microbiology, IGIMS, Patna, Bihar. Blood, urine, respiratory samples, pus, etc. were collected from all 105 patients to examine for any kind of bacterial infection.

The antibiotic resistance profile of isolates was analyzed between COVID-19-positive patients and non-COVID-19 patients by collecting randomly and retrospectively the same number of bacterial isolates, and the antibiotic resistance profile of non-COVID patients was compared with those of COVID patients.

Pathogen identification

All clinical specimens were processed and cultured on different media to support the growth of the bacteria as per our standard operating procedures (SOP) of Bacteriology. Identification and characterization of isolates were performed based on Gram staining [13], microscopic characteristics, colony characteristics, and biochemical tests [14] using standard microbiological methods and were further confirmed by MALDI-TOF MS (matrix-assisted laser desorption/ionization-time of flight mass spectrometry, BioMérieux, France).

Antibiotic sensitivity testing (AST)

AST of clinical isolates was carried out by the Kirby-Bauer disk diffusion method based on the Clinical and Laboratory Standards Institute 2020 recommendations [15]. The antibacterial drugs tested for gram-negative bacteria included amikacin, amoxicillin, ampicillin, cefepime, ceftriaxone, ciprofloxacin, imipenem, meropenem, nitrofurantoin, gentamicin, ertapenem, piperacillin/tazobactam, and trimethoprim/sulfamethoxazole. Colistin MIC was determined using the broth microdilution method. The antibacterial drugs against gram-positive pathogens included vancomycin, ciprofloxacin, linezolid, ampicillin, gentamicin (high concentration), nitrofurantoin, tetracycline, and erythromycin. According to the Centers for Disease Control and Prevention, isolates having resistance to at least one agent in three or more different classes of antibiotics are classified as multidrug-resistant pathogens. Quality control procedures for microscopy and AST were followed by using the American Type Culture Collection strains *Staphylococcus aureus* 25923 and *Escherichia coli* 25922.

Clinical specimens obtained during the study period were processed in biosafety cabinets using approved personal protective equipment. All samples were disposed of according to the Indian biomedical waste disposal guidelines.

Data analysis

Templates were generated in an Excel sheet (Microsoft, Washington, USA) and analysis of data was done using SPSS Statistics version 27 (IBM Corp. Released 2020. IBM SPSS Statistics for Windows, Version 27.0. Armonk, NY: IBM Corp.). The number of patients, number of isolates, and number of resistant organisms is presented as n. Patient age is presented as mean ± standard deviation (SD). The study was approved by the IGIMS, Patna, Institutional Ethics Committee (166/IEC/IGIMS/2021).

## Results

**Figure 1:**
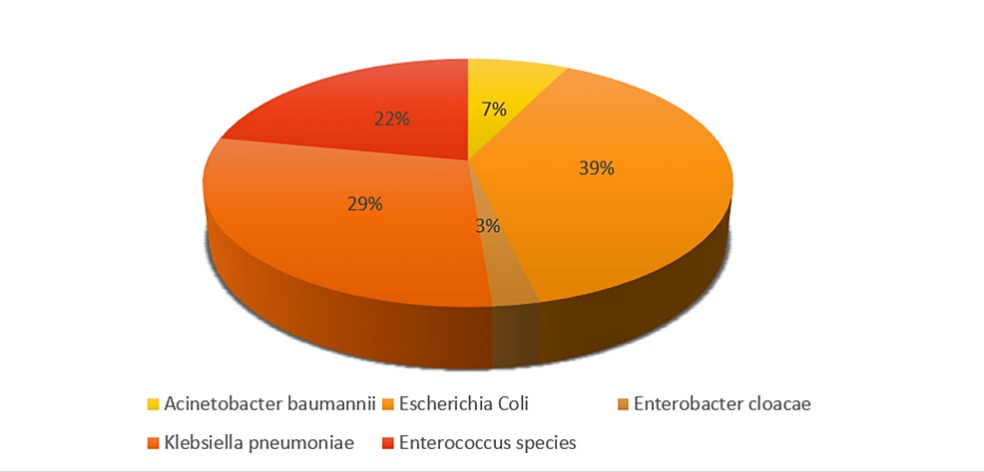
Distribution of microbial pathogen isolated from COVID-19 patients (n=41)

A total of 105 hospitalized COVID-19-positive patients samples were received during the study period from February 2021 to July 2021. It was seen that the mean age of the study participants was 51.57 ± 14.76 years, and males (66.7%, 70/105) were more than females (33.3%, 35/105) (Table 1). The majority of the patients were 91 out of 105 (86.67%) from the ward and 14 from the ICU (13.33%). Of the total samples tested, 62 (59%) were urine samples, 26 (24.8%) were respiratory specimens, 13 (12.4%) were pus samples, 3 (2.9%) were body fluids, and 1 (1%) were tissue sample (Table 1). The predominant sample received during the study period was urine in 62 out of 105 (59%) of the patients. A total of 105 samples were received for microbiological culture, of which 48 (45.71%) samples were culture-negative or sterile. Overall, 57 (54.28%) samples were found to be culture positive, including those categorized as contaminants. Among the total pathogen isolates (n=57) obtained from patients with SARS-CoV-2 admitted to the ward and ICU, 56.14% (32) were gram-negative, 26.31% (15) were *Candida*, and 17.54% (10) were gram-positive pathogens (Table 1). Of the total 57 organisms isolated, one was classified as a contaminant (coagulase-negative *Staphylococcus*), and 15 isolated were excluded as they were found to be fungal growth (*Candida*). Of the pathogens isolated, gram-negative bacteria were the predominant pathogen (78.04%, 32/41). The most isolated pathogen was *Escherichia coli* (39.02%, 16/41), followed by *Klebsiella pneumoniae* (29.26%, 12/41), *Acinetobacter baumannii* (7.31%, 3/41), and *Enterobacter cloacae* (2.43%, 1/41). *Enterococcus* spp. as gram-positive bacteria were isolated in 21.95% (9/41) of patients (Figure 1).

**Table 1:** Background characteristics of the COVID-19 patients assessed for pathogenic isolates (n=105) #Mean (+ Std deviation), BYLC: budding yeast-like cell, CONS: coagulase-negative *Staphylococcus*, NG: no growth

Background characteristics	Frequency (n)	Proportion (%)
Age	51.57 (+ 14.76)^ #^	
Gender		
Male	70	66.7
Female	35	33.3
Admission in:		
Ward	91	86.67
ICU	14	13.33
Site of sample:		
Urine	62	59
Respiratory sample	26	24.8
Pus	13	12.4
Body fluid	03	2.9
Tissue	01	1.0
Pathogenic isolates:		
Acinetobacter baumannii	3	2.9
BYLC	15	14.3
Escherichia coli	16	15.2
Enterobacter cloacae	1	1.0
Klebsiella pneumoniae	12	11.42
*Enterococcus* spp.	09	8.6
CONS	1	1
NG	48	45.7

The most common site of isolation of pathogen was from urine samples (36/62), followed by respiratory samples (16/26) and pus (5/13). Significant growth was seen in 36/62 urine cultures, with *Escherichia coli* (8/36, 22.22%) and *Enterococcus* spp. (8/36,22.22%) being the predominant pathogens. Fifteen out of 36 shows BYLC growth which was excluded and 26/62 showed insignificant/no growth.

Of the 26 respiratory samples, 16 (61.53%) were culture positive. *Klebsiella pneumoniae* (9/16, 50%) was the predominant pathogen followed by *Escherichia coli* (5/16, 31.25%).

Positive pus culture was found in 5/13 (38.46%) samples, and 1/5 (20%) were classified as contaminants. *Escherichia coli* (3/4,75%) and *Enterobacter cloacae* (1/4,25%) were the commonly isolated pathogens.

Table 2 depicts the association between bacteriological isolates and the site of the sample taken in COVID-19 patients, where there is a significant association (p = 0.018) between the type of sample collection and the bacteria isolated from the sample. The urine sample (62) yielded a maximum number of BYLC (24.2%), followed by *Escherichia coli* (12.9%).

**Table 2:** An overview of the bacteriological isolates from various clinical samples taken from COVID-19 patients (n=105) BYLC: budding yeast-like cell, CONS: coagulase-negative *Staphylococcus*,* *NG: no growth

Samples	Total	Bacteriological Isolates									
		*Acinetobacter baumannii* n (%)	BYLC n (%)	CONS n (%)	*Escherichia coli *n (%)	*Enterobacter cloacae *n (%)	*Enterococcus* spp. n (%)	*Klebsiella pneumoniae *n (%)	NG n (%)	Chi-square (df)	p-value
Body fluid	3	0	0	0	0	0	0	0	03 (100)	50.985 (32)	0.018
Pus	13	0	0	01 (7.7)	03 (23.1)	01 (7.7)	0	0	08 (61.5)		
Respiratory sample	26	01 (3.8)	0	0	05 (19.2)	0	01 (3.8)	09 (34.61)	10 (38.5)		
Tissue	1	0	0	0	0	0	0	0	01 (100)		
Urine	62	02 (3.2)	15 (24.2)	0	08 (12.9)	0	08 (12.9)	03 (4.8)	26 (41.9)		

Antibiotic resistance profile of isolated pathogens

The antibiotic resistance pattern of gram-positive and gram-negative bacteria (*Enterobacterales* and non-*Enterobacterales*) among COVID-19 patients is shown in Table 3. Among the gram-negative bacteria (*Enterobacterales*), the highest resistance was seen in ampicillin (100%, 29/29), followed by ciprofloxacin (96.6%, 28/29), ceftriaxone (93.1%, 27/29), cefepime (86.2%, 25/29), trimethoprim/sulfamethoxazole (82.8%, 24/29), amoxicillin (75.9%, 22/29), and piperacillin/tazobactam (72.4%, 21/29). Overall resistance to carbapenems was found to be 72.4%. All isolates were sensitive to colistin (100%). An intermediate resistance pattern was seen for antibiotics like ceftriaxone (3.4%), trimethoprim/sulfamethoxazole (3.4%), ertapenem (3.4%), and meropenem (3.4%). For non-*Enterobacterales,* the highest resistance was seen in ceftriaxone (66.66%,2/3), followed by gentamicin, imipenem, and trimethoprim/sulfamethoxazole which was 33.33%. Among the gram-positive pathogen isolated was *Enterococcus* spp. which showed maximum resistance for ciprofloxacin, and gentamicin (high-level) was 100% (9/9), followed by ampicillin and tetracycline which was 88.9% (8/9). About 44.44% (4/9) were vancomycin-resistant. All *Enterococcus* spp. were sensitive to linezolid (100%).

**Table 3:** Distribution of antibiotic resistance pattern among COVID-19 patients

Antibiotics	Resistant [n (%)]	Sensitive [n (%)]	Intermediate [n (%)]
Resistance pattern among gram-negative bacteria (*Enterobacterales*) (n=29)			
Ampicillin	29 (100)	--	--
Gentamicin	21 (72.41)	8 (36.4)	--
Ciprofloxacin	28 (96.6)	1 (3.4)	--
Cefepime	25 (86.2)	4 (13.8)	--
Ceftriaxone	27 (93.1)	1 (3.4)	01 (3.4)
trimethoprim/sulfamethoxazole	24 (82.8)	4 (13.8)	01 (3.4)
Imipenem	21 (72.4)	8 (27.6)	--
Piperacillin/tazobactam	21 (72.4)	8 (27.6)	--
Amoxicillin	22 (75.9)	7 (24.1)	--
Ertapenem	21 (72.4)	7 (24.1)	01 (3.4)
Meropenem	21 (72.4)	7(24.1)	01 (3.4)
Amikacin	12 (41.4)	17 (58.6)	--
Colistin	00	29 (100)	--
Nitrofurantoin	5 (45.5)	24 (82.75)	--
Resistance pattern among gram-negative bacteria (non-*Enterobacterales*) (n=03)			
Amoxicillin	00	03 (100)	--
Gentamicin	01 (33.33)	02 (66.66)	--
Ciprofloxacin	00	03 (100)	--
Imipenem	01 (33.33)	02 (66.66)	--
Ceftriaxone	02 (66.66)	01 (33.33)	--
trimethoprim/sulfamethoxazole	01 (33.33)	02 (66.66)	--
Meropenem	00	03 (100)	--
Piperacillin/tazobactam	00	03 (100)	--
Resistance pattern among gram-positive bacteria (n=9)			
Ampicillin	08 (88.9)	01 (11.1)	--
Ciprofloxacin	09 (100)	00	--
Gentamicin (high-level)	09 (100)	00	--
Nitrofurantoin	06 (66.66)	3 (33.33)	--
Linezolid	00	09 (100)	--
Vancomycin	04 (44.44)	05 (55.55)	--
Tetracycline	08 (88.9)	01 (11.1)	--

The association between different bacteriological isolates and their antibiotic resistance pattern among COVID-19 patients is depicted in Table 4, where the resistance of *Klebsiella pneumoniae* and *Escherichia coli* to piperacillin/tazobactam was significantly associated (p = 0.026).

**Table 4:** Association between different bacteriological isolates and their antibiotic resistance pattern of gram-negative bacteria in COVID-19 patients [*Fisher’s exact-], NR: not recommended according to the Clinical and Laboratory Standards Institute

Resistance to following antibiotics	*Escherichia coli* (n=16)	*Klebsiella pneumoniae* (n=12)	*Enterobacter cloacae* (n=1)	*Acinetobacter baumannii* (n=03)	p-value
Ampicillin	16	NR	01	00	--
Gentamicin	11	10	00	01	1^*^
Ciprofloxacin	16	12	00	00	0.069
Cefepime	15	10	00	NR	0.164
Ceftriaxone	16	11	00	02	0.704
trimethoprim/sulfamethoxazole	15	09	00	01	0.271
Imipenem	10	11	00	01	0.139
Piperacillin/tazobactam	12	09	00	00	0.026
Amoxicillin	13	09	00	00	0.135
Ertapenem	11	10	00	NR	0.068
Meropenem	11	10	00	00	0.138
Amikacin	02	10	00	NR	1
Colistin	00	00	00	00	--
Nitrofurantoin	03	02	00	00	0.545

The antibiotic resistance profile of isolates was analyzed between COVID-19-positive patients and non-COVID-19 patients by collecting randomly and retrospectively the same number of bacterial isolates, and the antibiotic resistance profile of non-COVID patients was compared with that of COVID patients.

**Figure 2:**
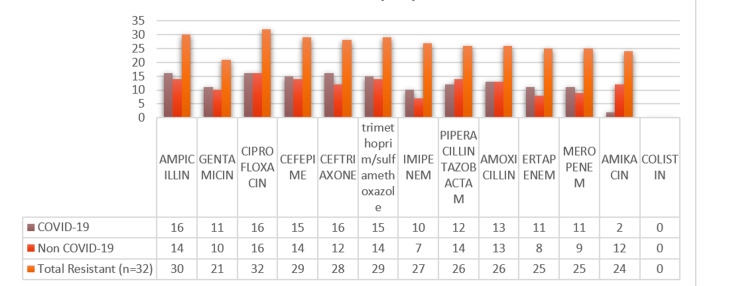
Escherichia coli antibiotic resistance profile among COVID-19 (n=16) and non-COVID-19 patients (n=16)

The antibiotic resistance pattern of *Escherichia coli *(n=32) of COVID-19 (n=16/32) patients and non-COVID (n=16/32) patients is presented in Figure 2. Among the 16 strains of *Escherichia coli* from COVID patients, the highest resistance was seen in antibiotics like ampicillin (100%), ceftriaxone (100%), ciprofloxacin (100%), trimethoprim/sulfamethoxazole (93.75%), cefepime (93.75%), amoxicillin (81.25%), piperacillin/tazobactam (75%), gentamicin (68.75%), ertapenem (68.75%), and imipenem (62.5%).

Therefore, the total resistance pattern of *Escherichia coli* from both COVID and non-COVID patients is as follows: 100% (32/32) of the strains showed resistance to ciprofloxacin, 93.75% (30/32) to ampicillin, 90.62% (29/32) to cefepime and trimethoprim/sulfamethoxazole, 87.5% (28/32) to ceftriaxone, 81.25% (26/32) to piperacillin/tazobactam and amoxicillin, 78.12% (15/32) to ertapenem and meropenem, 65.62% (21/32) to gentamicin, and 43.75% (14/32) to amikacin.

**Figure 3:**
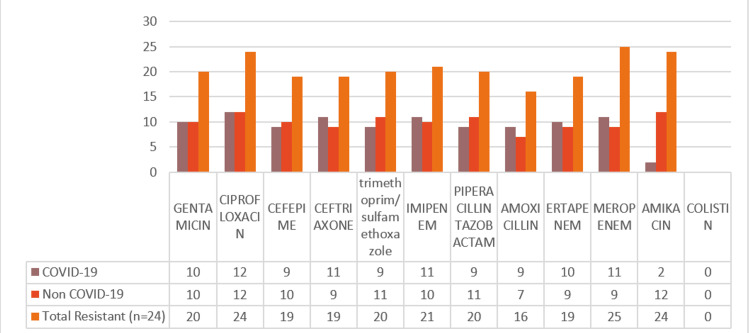
Klebsiella pneumoniae antibiotic resistance profile among COVID-19 (n=12) and non-COVID-19 patients (n=12)

The antibiotic resistance pattern of *Klebsiella pneumoniae* (n=24) of COVID-19 (n=12/24) patients and non-COVID (n=12/24) patients are presented in Figure 3. Among the 12 strains of *klebsiella pneumoniae* from COVID patients, the highest resistance was seen 100% (12) in ciprofloxacin, 91.66% (11) in ceftriaxone and imipenem, 83.33% (10) in cefepime, amikacin, gentamicin, meropenem, and ertapenem, 75% (9) in amoxicillin, piperacillin/tazobactam, and trimethoprim/sulfamethoxazole.

The total resistance pattern of *Klebsiella pneumoniae* isolates from both COVID and non-COVID patients is as follows: 100% (24/24) of the strains showed resistance to ciprofloxacin, 87.5% (11/24) to imipenem, 83.33% (20/24) to gentamicin, ceftriaxone, and trimethoprim/sulfamethoxazole, 79.16% (19/24) to piperacillin/tazobactam, cefepime, ertapenem, and meropenem, 75% (18/24) to amikacin, and 66.66% (16/24) to amoxicillin.

**Figure 4:**
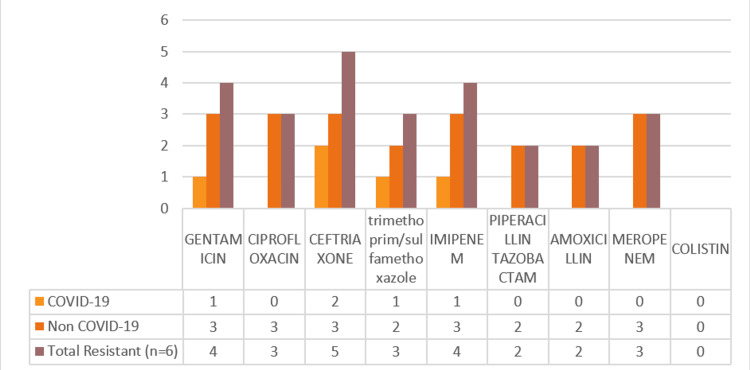
Acinetobacter baumannii antibiotic resistance profile among COVID-19 (n=3) and non-COVID-19 patients (n=3)

Also, the resistance profile of *Acinetobacter baumannii* (n=6) was analyzed. Of these, n=3/6 isolates from COVID-19 patients and n= 3/6 from non-COVID-19 patients isolates from COVID-19 (n=3) patients (Figure 4). Among the three strains of *Acinetobacter baumannii* from COVID patients, the highest resistance was seen at 66.66% (2) % in ceftriaxone and 33.33% (1) in trimethoprim/sulfamethoxazole, gentamicin, and Imipenem. No resistance (0%) was seen in ciprofloxacin, meropenem, amoxicillin, and piperacillin/tazobactam.

The total resistance pattern of *Acinetobacter baumannii* isolates from both COVID and non-COVID patients is as follows: 83.33% (5/6) of the strains showed resistance to ceftriaxone, 66.66% (4/6) to gentamicin and imipenem, 50% (3/6) to ciprofloxacin, trimethoprim/sulfamethoxazole, and meropenem, and 33.33% (2/6) to piperacillin/tazobactam and amoxicillin.

**Figure 5:**
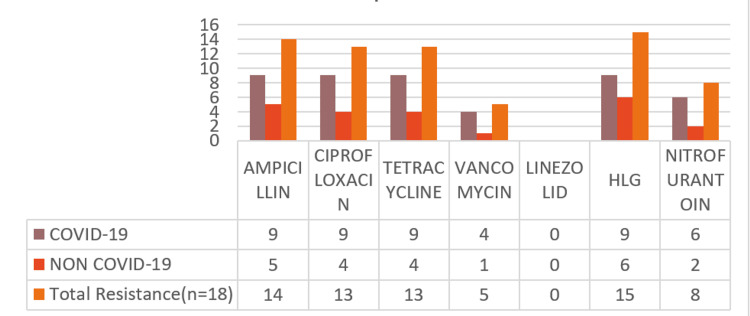
Enterococcus spp. antibiotic resistance profile among COVID-19 (n=9) and non-COVID-19 patients (n=9)

In this study, the antibiotic resistance pattern of *Enterococcus* spp. (n=18) of COVID-19 (n=9/24) patients and non-COVID (n=9/24) patients is presented in Figure 5. Among the nine strains of *Enterococcus* spp. from COVID patients, the highest resistance was seen at 100% (9) in ampicillin, ciprofloxacin, gentamicin (high level), and tetracycline, 66.66% (6) in nitrofurantoin, 44.44% in vancomycin, and no resistance was seen in linezolid.

Therefore, the total resistance pattern of *Enterococcus* spp. from both COVID and non-COVID patients is as follows: 83.33% (15/18) to gentamicin (high level), 77.77% (14/18) to ampicillin, 72.22% (13/18) to ciprofloxacin and tetracycline, 44.44% (8/18) to nitrofurantoin, 27.77% (5/18) to vancomycin, and 0% (0/18) to linezolid.

## Discussion

The COVID-19 pandemic not only resulted in severe economic losses but also posed major challenges to healthcare. The whole world also witnessed the uncontrolled/overuse of broad-spectrum antimicrobials drug with multiple drug combinations administered to patients in wards and ICUs. The fear of COVID-19 infection and the reluctance of healthcare workers to enter the COVID-19 ICU and wards led to a complete breakdown and unintentional neglect of the healthcare system, ultimately jeopardizing the established antimicrobial stewardship policy program. Bacterial coinfections occurring in hospitalized patients with COVID-19 are a major cause of mortality. According to Langford et al.'s meta-analysis, COVID-19-infected ICU patients are at a higher risk of having bacterial co-infections than similarly ill patients from other ICU settings [7]. In a study conducted in Wuhan, of a total of 41 patients, secondary infections were reported in 31% of ICU patients and 10% of patients overall [16]. In the present study, we found that of the total 105 patients, 14 (13.33%) were ICU patients, and a total of 105 samples were received for microbiological culture, of which 48 (45.71%) samples were culture-negative or sterile. Overall, 57 (54.28%) samples were found to be culture positive, including those categorized as contaminants.

In our study, respiratory samples taken from COVID-19 patients remain predominant, and lower respiratory tract bacterial co-infections remain common in these patients. The underlying pathogenicity of bacterial coinfection in severe and critical cases of COVID-19 is due to host-pathogen interactions; these include pathogen virulence factors, immune response dysregulation, and disturbed microbial flora during viral pneumonia [17,18].

Of the pathogens isolated, gram-negative bacteria were the predominant pathogen (78.04%, 32/41). The most isolated pathogen was *Escherichia coli* (39.02%, 16/41), followed by *Klebsiella pneumoniae* (29.26%, 12/41), *Acinetobacter baumannii* (7.31%, 3/41), and *Enterobacter cloacae* (2.43%, 1/41). *Enterococcus* spp. as gram-positive bacteria were isolated in 21.95% (9/41) of patients. According to a recent study from China, gram-negative bacteria accounted for the majority of bacterial pathogens isolated from respiratory tract samples (26, 65%), and gram-positive bacteria accounted for the remainder (14, 34.99%). In this study, the most common bacterial pathogens were *Klebsiella pneumoniae* (n=11) and *Enterococcus faecium* (n=9), followed by *Acinetobacter baumannii* (n=8) [19]. Differences in the colonization of bacterial types between centers, clinical profiles of patients, and prophylactic antibiotic administration may explain the discrepancies between this study and the previous report.

As reported by Russell et al., gram-negative bacteria associated with bloodstream infections commonly belong to the Enterobacteriaceae family, which includes *Escherichia coli* [20]. In our study, *Klebsiella pneumoniae* was the predominant isolate causing respiratory tract infections affected by COVID-19, and *Escherichia coli* and *Enterococcus* spp. were the predominant isolates causing UTI. Among COVID-19 patients admitted to an ICU setting, *Klebsiella* spp. was frequently found to complicate treatment options, according to a study by Sreenath et al. [21]. Another study by Arcari et al. showed that multidrug-resistant *Klebsiella* spp. remain a common pathogen in critically ill COVID-19 patients [22].

In COVID-19 patients, among the gram-negative bacteria (*Enterobacterales*), the highest resistance was seen in ampicillin (100%, 29/29), followed by ciprofloxacin (96.6%, 28/29), ceftriaxone (93.1%, 27/29), cefepime (86.2%, 25/29), trimethoprim/sulfamethoxazole (82.8%, 24/29), amoxicillin (75.9%, 22/29), and piperacillin/tazobactam (72.4%, 21/29). Overall resistance to carbapenems was found to be 72.4%. All isolates were sensitive to colistin (100%). A study by Mahmoudi et al. showed that *Escherichia coli* from COVID-19 patients has greater resistance to cephalosporins, co-trimoxazole, and piperacillin/tazobactam [23].

Amongst the urinary isolates, *Escherichia coli* and *Enterococcus* spp. remained the most common pathogen followed by *Klebsiella pneumoniae* in the urine samples of the COVID patients. A switch in distribution occurred in 2020 when gram-negative isolates rose from 69% to 82%. Among the gram-positive pathogen isolates, *Enterococcus* spp. has shown that the maximum resistance to ciprofloxacin and gentamicin (high level) was 100% (9/9), followed by ampicillin and tetracycline which was 88.9% (8/9). About 44.44% (4/9) were vancomycin-resistant.

Fluoroquinolones, cephalosporins, carbapenems, and vancomycin, which belong to the AWaRe classification of drugs by the WHO, were the most used intravenous antimicrobial agents in our study. Comparing the pandemic with the pre-COVID-19 year, Clancy et al. observed an increase in AMR during the pandemic and [24] emphasized that antibiotics overuse leads to an abundant opportunity for bacterial pathogens to gradually develop mechanisms to acquire drug/antibiotic resistance [25-27].

Enterobacterales were significantly more resistant to drugs like fluoroquinolones, cephalosporin, and carbapenem in COVID-19 patients compared to non-COVID-19 patients. In a recent study from Egypt, gram-negative isolates were mostly extended-spectrum beta-lactamases or carbapenemase producers which are similar to our study [28].

The resistance profile of *Acinetobacter baumannii* in our study was lower as compared to non-COVID- 19 patients. This finding is not concordant with the study done by Li et al. [8]. However, the study conducted by Mahmoudi [23] and another study conducted by Khurana et al. [29] showed higher resistance among *Klebsiella* spp., followed by *Acinetobacter* spp. In our study, *Klebsiella* spp. showed higher resistance followed by *Escherichia coli*. A study by Sharifipour et al. showed that widespread resistance was also contributed by *Acinetobacter* spp. [30]. The disparity may be due to the difference in the drug resistance profiles between nations and/or local hygiene measures implemented for patients with COVID-19.

The present study has some limitations. First, the comprehensive clinical data and severity of the disease of these patients were not studied. The second reason is that our study, which was conducted at one hospital, did not encompass a large number of patients. In spite of the absence of data on clinical severity, the study focused on describing the prevalence of microorganisms and AST outcomes in individuals with COVID-19 as a whole. In addition, the sample size covers a period of six months in the pandemic period. Compared to other studies, this study compared both the prevalence of bacterial infections and AMR patterns in patients with and without COVID-19.

## Conclusions

In conclusion, we found 39% of bacterial infections in COVID-19 patients; the most common infection was urine infection, and an increase in antibiotic resistance was seen more in COVID-19 patients as compared with non-COVID-19 patients. An emerging virus, COVID-19, is known as a new disease. It is a major medical concern that viral-bacterial co-infections result in higher mortality rates. There is a possibility that stricter antimicrobial handling protocols in COVID-19 management plans can prevent the development of AMR. To reduce inappropriate antibiotic prescribing for COVID-19 patients, clinicians should have access to descriptive data on antimicrobial susceptibility. The severity of infections should be monitored with serum biomarkers before any empiric antimicrobials are administered. Regular prescribing reviews, followed by ongoing monitoring of hospital infection control practices by the hospital clinical infection control team and clinician education, can not only improve the quality of medications in the long term but can also help us to combat the threat of AMR.

## References

[1] Zu ZY, Jiang MD, Xu PP, Chen W, Ni QQ, Lu GM, Zhang LJ (2020). Coronavirus disease 2019 (COVID-19): a perspective from China. Radiology.

[2] Möhlenkamp S, Thiele H (2020). Ventilation of COVID-19 patients in intensive care units. Herz.

[3] Zhu N, Zhang D, Wang W (2020). A novel coronavirus from patients with pneumonia in China, 2019. N Engl J Med.

[4] Zhou F, Yu T, Du R (2020). Clinical course and risk factors for mortality of adult in patients with COVID-19 in Wuhan, China: a retrospective cohort study. Lancet.

[5] Phelan AL, Katz R, Gostin LO (2020). The novel coronavirus originating in Wuhan, China: challenges for global health governance. JAMA.

[6] Garcia-Vidal C, Sanjuan G, Moreno-García E (2021). Incidence of co-infections and superinfections in hospitalized patients with COVID-19: a retrospective cohort study. Clin Microbiol Infect.

[7] Langford BJ, So M, Raybardhan S (2020). Bacterial co-infection and secondary infection in patients with COVID-19: a living rapid review and meta-analysis. Clin Microbiol Infect.

[8] Li J, Wang J, Yang Y, Cai P, Cao J, Cai X, Zhang Y (2020). Etiology and antimicrobial resistance of secondary bacterial infections in patients hospitalized with COVID-19 in Wuhan, China: a retrospective analysis. Antimicrob Resist Infect Control.

[9] Gautret P, Lagier JC, Parola P (2020). Hydroxychloroquine and azithromycin as a treatment of COVID-19: results of an open-label non-randomized clinical trial. Int J Antimicrob Agents.

[10] Rawson TM, Moore LS, Zhu N (2020). Bacterial and fungal coinfection in individuals with coronavirus: a rapid review to support COVID-19 antimicrobial prescribing. Clin Infect Dis.

[11] Lai CC, Wang CY, Hsueh PR (2020). Co-infections among patients with COVID-19: the need for combination therapy with non-anti-SARS-CoV-2 agents?. J Microbiol Immunol Infect.

[12] Jancin B (2020). Secondary infections common in COVID-19, implications unclear. Mdedge-Intern News.

[13] Mackie TJ, McCartney JE, Collee JG (1996). Staining methods. Mackie & McCartney practical medical microbiology.

[14] Mackie TJ, McCartney JE, Collee JG (1996). Test for identification of bacteria. Mackie & McCartney practical medical microbiology.

[15] (2020). Performance standards for antimicrobial susceptibility testing. https://clsi.org/standards/products/microbiology/documents/m100/.

[16] Joo KW, Gabrielle L, Matthew Z, Harleen K, Magbual N (2020). Superinfections and coinfections in COVID-19—separating the signal from the noise. Intern Med. News free C. Online.

[17] Lucien MA, Canarie MF, Kilgore PE (2021). Antibiotics and antimicrobial resistance in the COVID-19 era: perspective from resource-limited settings. Int J Infect Dis.

[18] Vaillancourt M, Jorth P (2020). The unrecognized threat of secondary bacterial infections with COVID-19. mBio.

[19] Zhang H, Zhang Y, Wu J (2020). Risks and features of secondary infections in severe and critical ill COVID-19 patients. Emerg Microbes Infect.

[20] Russell CD, Fairfield CJ, Drake TM (2021). Co-infections, secondary infections, and antimicrobial use in patients hospitalised with COVID-19 during the first pandemic wave from the ISARIC WHO CCP-UK study: a multicentre, prospective cohort study. Lancet Microbe.

[21] Sreenath K, Batra P, Vinayaraj EV (2021). Coinfections with other respiratory pathogens among patients with COVID-19. Microbiol Spectr.

[22] Arcari G, Raponi G, Sacco F (2021). Klebsiella pneumoniae infections in COVID-19 patients: a 2-month retrospective analysis in an Italian hospital. Int J Antimicrob Agents.

[23] Mahmoudi H (2020). Bacterial co-infections and antibiotic resistance in patients with COVID-19. GMS Hyg Infect Control.

[24] Clancy CJ, Nguyen MH (2020). Coronavirus disease 2019, superinfections, and antimicrobial development: what can we expect?. Clin Infect Dis.

[25] Cavalcanti AB, Zampieri FG, Rosa RG (2020). Hydroxychloroquine with or without azithromycin in mild-to-moderate Covid-19. N Engl J Med.

[26] Fiolet T, Guihur A, Rebeaud ME, Mulot M, Peiffer-Smadja N, Mahamat-Saleh Y (2021). Effect of hydroxychloroquine with or without azithromycin on the mortality of coronavirus disease 2019 (COVID-19) patients: a systematic review and meta-analysis. Clin Microbiol Infect.

[27] Wang Z, Yang B, Li Q, Wen L, Zhang R (2020). Clinical features of 69 cases with coronavirus disease 2019 in Wuhan, China. Clin Infect Dis.

[28] Ramadan HK, Mahmoud MA, Aburahma MZ (2020). Predictors of severity and co-infection resistance profile in COVID-19 patients: first report from upper Egypt. Infect Drug Resist.

[29] Khurana S, Singh P, Sharad N (2021). Profile of co-infections &amp; secondary infections in COVID-19 patients at a dedicated COVID-19 facility of a tertiary care Indian hospital: implication on antimicrobial resistance. Indian J Med Microbiol.

[30] Sharifipour E, Shams S, Esmkhani M (2020). Evaluation of bacterial co-infections of the respiratory tract in COVID-19 patients admitted to ICU. BMC Infect Dis.

